# Rheumatism—Lac. Caninum

**Published:** 1882-12

**Authors:** Clarence Willard Butler

**Affiliations:** Montclair, N. J.


### RHEUMATISM—LAC CANINUM.

Clarence Willard Butler, Montclair, N. J.

At 10 A. M., December 12th, 1880, I was called to see Mrs. J., a large, plethoric woman, fifty years of age, and of irregularly intemperate habits, a periodical tippler. She and her husband,

> "Two souls with but a single thought,
> Two hearts that beat as one,"

got drunk together on the 10th of the month, and while sleeping off her intoxication that night the weather changed suddenly colder, and the fire, neglected, went entirely out. She awoke toward morning thoroughly chilled, and the next day suffered with fever and "pains in all her bones." She was in bed, on occasion of my visit, with the joints of her left hand, her left ankle, and left knee extremely painful, swollen moderately, and slightly red. The day before, in addition to the bone pains already mentioned, she had less severe but similar swellings and sufferings in the right ankle and right knee. The pain was sharp and darting and aggravated by any motion. She had many pains coming and going, and appearing now here and now there. Temperature, $102\frac{4}{5}$. Pulse, rapid and quick—count not recorded. Thirst. ℞. Pulsatilla$^{200}$ every hour, a dose of an aqueous solution.

December 13th, at 10 A. M., the left ankle and knee relieved, but the right shoulder and elbow similarly affected though in slighter degree. The other joints unchanged in appearance. Pulse still rapid and quick. Temperature, 103. Thirst. No appetite. Tongue relaxed, flabby, with whitish coating. Bowels constipated. Had perspired considerably through the night, the perspiration having a "rank" smell. ℞. Lac Caninum$^{cm}$. (Swan), one dose dry on the tongue. Sac. Lac.

December 14th, at 11 A. M., she was much improved in *every* respect. Temperature, 100$\frac{2}{5}$. ℞. Sac. Lac.

December 15th, at 3 P. M., I found her sitting up, all the pain and swelling gone, and, in fact, no appearance or sensation of rheumatism. She was weak and tremulous, but otherwise well. No medicine. Discharged.

January 15th, 1881, she reported no return of the rheumatism or further illness of any kind.

